# Calibration and Localization of Optically Pumped Magnetometers Using Electromagnetic Coils

**DOI:** 10.3390/s22083059

**Published:** 2022-04-15

**Authors:** Joonas Iivanainen, Amir Borna, Rasmus Zetter, Tony R. Carter, Julia M. Stephen, Jim McKay, Lauri Parkkonen, Samu Taulu, Peter D. D. Schwindt

**Affiliations:** 1 Sandia National Laboratories, Albuquerque, NM 87185, USA; aborna@sandia.gov (A.B.); tcarter@sandia.gov (T.R.C.); pschwin@sandia.gov (P.D.D.S.); 2 Department of Neuroscience and Biomedical Engineering, Aalto University School of Science, FI-00076 Aalto, Finland; rasmus.zetter@aalto.fi (R.Z.); lauri.parkkonen@aalto.fi (L.P.); 3 Mind Research Network a Division of Lovelace Biomedical Research Institute, Albuquerque, NM 87106, USA; jstephen@mrn.org; 4 Candoo Systems Inc., Port Coquitlam, BC V3C 5M2, Canada; jim.mckay@candoosys.com; 5 University of Washington, Seattle, WA 98195, USA; staulu@uw.edu

**Keywords:** calibration, sensor localization, co-registration, optically pumped magnetometer, magnetoencephalography, on-scalp MEG, fluxgate magnetometer, electromagnetic coil

## Abstract

In this paper, we propose a method to estimate the position, orientation, and gain of a magnetic field sensor using a set of (large) electromagnetic coils. We apply the method for calibrating an array of optically pumped magnetometers (OPMs) for magnetoencephalography (MEG). We first measure the magnetic fields of the coils at multiple known positions using a well-calibrated triaxial magnetometer, and model these discreetly sampled fields using vector spherical harmonics (VSH) functions. We then localize and calibrate an OPM by minimizing the sum of squared errors between the model signals and the OPM responses to the coil fields. We show that by using homogeneous and first-order gradient fields, the OPM sensor parameters (gain, position, and orientation) can be obtained from a set of linear equations with pseudo-inverses of two matrices. The currents that should be applied to the coils for approximating these low-order field components can be determined based on the VSH models. Computationally simple initial estimates of the OPM sensor parameters follow. As a first test of the method, we placed a fluxgate magnetometer at multiple positions and estimated the RMS position, orientation, and gain errors of the method to be 1.0 mm, 0.2°, and 0.8%, respectively. Lastly, we calibrated a 48-channel OPM array. The accuracy of the OPM calibration was tested by using the OPM array to localize magnetic dipoles in a phantom, which resulted in an average dipole position error of 3.3 mm. The results demonstrate the feasibility of using electromagnetic coils to calibrate and localize OPMs for MEG.

## 1. Introduction

Magnetoencephalography (MEG) is a functional neuroimaging technique in which the magnetic fields of electrically active neuron populations in the human brain are detected outside of the head [1]. To estimate the active neural sources in the brain from the measured field patterns, an inverse problem must be solved, which involves modeling of the electric neural sources and their magnetic fields as detected by the MEG sensor array. To make the modeling and the source estimation precise, the sensor geometry must be accurately co-registered with the magnetic resonance images of the subject’s head, i.e., precise knowledge of the sensor positions and orientations with respect to the subject’s brain is needed. In addition, the sensor gains (conversion factors from volts to tesla) must be known.

Traditional MEG systems utilize superconducting quantum interference devices (SQUIDs) that are housed inside a Dewar in a rigid, helmet-shaped configuration. To calibrate the SQUID sensors, two types of methods have been used. In the first group of methods, large Helmholtz coils producing uniform magnetic fields are employed to find the SQUID gains (e.g., [2,3]), while in the second group, small individual calibration coils are used to estimate the SQUID positions, orientations, and gains [4,5,6]. The rigid transformation between the SQUID sensor positions and the subject’s head is usually obtained by localizing small coils attached to the subject’s scalp by digitizing the coil positions relative to known fiducial points before the measurement (e.g., [7,8]).

Recently, a new type of magnetometer with sensitivity suitable for MEG has become available. In contrast to SQUIDs, which detect the field at least ~2 cm from the scalp, these optically pumped magnetometers (OPMs) can measure the magnetic field within millimeters from the scalp, increasing the signal magnitude and the spatial resolution of the measurement [9,10]. The introduction of this new sensor type has led to the development of various co-registration methods (e.g., [11]), with new requirements that must be taken into account. The possibility of obtaining higher spatial resolution due to the shorter measurement distance motivates the development of accurate methods to capture the finer spatial details of the magnetic fields that the OPMs may provide in comparison to the SQUID-based MEG systems [12]. Moreover, as OPM positions can be optimized for each subject in a flexible manner, the co-registration method should be capable of resolving the individual sensor locations and orientations on a per-subject basis.

Most of the co-registration methods developed for OPM–MEG systems have used optical scanners to measure the sensor positions with respect to the head [11,13,14,15]. These methods either co-register the sensor helmet with the brain and use the helmet geometry to obtain the individual sensor positions, or they directly identify the individual sensor positions from the optical scan. The optical methods have some drawbacks, as they can suffer from line-of-sight issues, and cannot necessarily obtain precise OPM positions and orientations, as these are difficult to obtain from geometric models (they depend not only on the external geometry of the OPM, but also on the OPM laser beam shape, the vapor cell optical depth and on-sensor coil configuration, etc.). Additionally, a magnetic method has been proposed that uses a set of pre-measurement digitized small dipolar coils attached to the subject’s head for finding the positions, orientations, and gains of the on-scalp MEG sensors with respect to the subject’s head/brain [16,17].

Here, we propose a method that uses large external electromagnetic coils to estimate the position, orientation, and gain of an OPM. The use of large electromagnetic coils for OPM calibration is attractive, as most of the OPM–MEG systems already employ large coils for magnetic field control (e.g., [18,19,20]); the proposed method does not then necessarily need any extra hardware. The method is based on modeling the coil fields using coefficients of vector spherical harmonic (VSH) expansions, which are obtained from *a priori* measurements of the coil fields. We used this method to find the positions, orientations, and gains of OPMs in a 48-channel sensor array. Subsequently, we localized magnetic dipoles of a phantom using the calibrated OPM array, and compared the localization results to the known locations of the dipoles.

## 2. Background

In this section, we lay out the methodology for obtaining the gain as well as the 3D position and orientation of a magnetic field sensor (hereafter referred to as *sensor parameters*) using magnetic fields generated by a set of *N* electromagnetic coils. In this section we present the method for a general magnetic field sensor; however, in the later sections, we apply the method for calibrating OPMs in a particular MEG system.

The basic idea behind the method is straightforward: We measure the sensor response when each coil is excited. We model the magnetic fields of the coils, and find the sensor parameters that minimize the sum of squared errors between the measurements and the models. More formally, we denote the measured sensor response of the *i*th coil as $y_{i}$. We model the sensor output (in volts) as follows:

(1) $$b_{i}=g{\vec{B}}_{i}\left(\vec{r}\right)\cdot \vec{n},$$

where g is the sensor gain (V/T), $\vec{r}$ is the sensor position, $\vec{n}$ is a unit vector representing the sensor orientation, and ${\vec{B}}_{i}\left(\vec{r}\right)$ is the modeled magnetic field of the *i*th coil at $\vec{r}$. We seek the sensor parameters by minimizing the sum of squared errors between the models and the measurements:

(2) $$\underset{g,\;\;\vec{r},\vec{n}\;}{\mathrm{argmin}}\overset{N}{\underset{i=1}{\sum }}{\left(y_{i}-g{\vec{B}}_{i}\left(\vec{r}\right)\cdot \vec{n}\right)}^{2}.$$

### 2.1. Obtaining Magnetic Field Models

Accurate determination of the sensor parameters with Equation (2) relies on accurate models of the magnetic fields. In principle, the magnetic fields could be calculated by using computational models of the coils. However, such an approach is likely to be inaccurate, as it is difficult to correctly model factors such as coil non-idealities and magnetic field interaction with the surroundings (especially prominent when the coils are inside a magnetic shield, causing polarization effects). Therefore, it is preferable to base the models on measurements of the coil magnetic fields. To obtain the models, the coil magnetic fields must be measured at multiple positions with a vector magnetometer whose parameters are known accurately.

As the parameter space of the optimization problem (Equation (2)) is continuous, the representation of the coil magnetic field must be continuous as well; the discrete measurements of the magnetic field should allow for an accurate interpolation of the field. For example, the field model could be obtained by a linear interpolation if the field is mapped using sufficiently dense three-axis measurements. Another option is to model the field as a linear combination of basis functions. When the basis function coefficients are known, the field can be interpolated. Here, we opt for the latter approach, and model the magnetic field using a linear combination of vector spherical harmonics (VSHs). Compared to other interpolation methods or basis function expressions, the VSH model has some advantages: First, when the coils are sufficiently far away from the sensor, their field energy will be in the lowest VSH orders; thus, only a relatively low number of VSH components is sufficient for modeling, as compared to models where such a clear hierarchical structure is not achievable. Second, because the expansion is based on the physical characteristics of magnetic fields (given by Maxwell’s equations), such an expansion offers some robustness against noise.

In a source-free space, the magnetic fields of far-away sources (the magnetic field coils) can be represented with VSHs (e.g., [21,22]) as follows:

(3) $${\vec{B}}_{i}\left(\vec{r}\right)=-\mu_{0}\overset{\infty }{\underset{l=1}{\sum }}\overset{l}{\underset{m=-l}{\sum }}\beta_{i}^{lm}r^{l-1}\sqrt{l\left(2l+1\right)}{\vec{W}}_{lm}\left(\theta ,\varphi \right)=\overset{\infty }{\underset{l=1}{\sum }}\overset{l}{\underset{m=-l}{\sum }}\beta_{i}^{lm}{\vec{w}}_{lm}\left(r,\theta ,\varphi \right),$$

where $\mu_{0}$ is the permeability of free space, l is the degree and m∈{−l,−l + 1,…,l − 1,l} is the order of the VSH ${\vec{W}}_{lm}$ (or ${\vec{w}}_{lm}$), (r,θ,φ) are the spherical coordinates, and $\beta_{i}^{lm}$ are the VSH coefficients. Equation (3) is valid inside a spherical volume that does not include the coils. The VSHs and their coefficients can be scaled arbitrarily; we opt for the latter expression in Equation (3) due to its simplicity. The VSHs corresponding to l = 1 are the three homogeneous components of the field along the Cartesian coordinate axes, while those corresponding to l = 2 are the five first-order gradients. Higher degrees correspond to higher-order gradients. The squared coefficients $\left\{\beta_{i}^{lm^{2}}\right\}$ comprise the VSH spectrum of the magnetic field.

The continuous field ${\vec{B}}_{i}\left(\vec{r}\right)$ can then be represented with a set of VSH coefficients that can be computed from the measurements of the coil field at known positions as follows (e.g., [23]):

(4) $$\beta_{i}=S^{†}m_{i},$$

where $\beta_{i}$ is a C×1 vector containing C VSH coefficients, S is an M×C matrix containing the ${\vec{w}}_{lm}$ patterns as detected by the M×1 measurements $m_{i}$, and $S^{†}$ denotes the pseudo-inverse of S.

### 2.2. Simplifying the Optimization Problem

Even if the obtained coil models were perfectly accurate, it is not guaranteed that a magnetic field sensor can be calibrated with this method, as the optimization of Equation (2) using the field models may not converge to the correct sensor parameters (~global optimum). Generally, the convergence will depend on the number of coils used and their VSH spectra. Next, we briefly describe how the optimization problem can be simplified.

If the coils generate low-order VSH components, the sensor parameters can be solved using linear equations. Specifically, we assume that $N_{H}$ coils generate homogeneous field components (linear combinations of VSHs corresponding to l = 1, m = −1, 0, 1), and that $N_{G}$ coils generate first-order gradients (l = 2, m=−2,−1, 0, 1, 2) and homogeneous components. As shown in the Appendix A, the sensor parameters can then be obtained as follows:

(5) $$g=H^{†}b_{H}$$

(6) $$r=G^{†}(b_{G}-H_{G}g),\;$$

where g=gn (the sensor orientation unit vector n multiplied by the sensor gain g), r is the sensor position vector, H is an $N_{H}\times 3$ matrix describing the uniform fields of the homogeneous coils, G is an $N_{G}\times 3$ matrix whose elements depend on g and the first-order gradients of the coils, $H_{G}$ is an $N_{G}\times 3$ matrix that contains the homogeneous components of the gradient fields, and $b_{H}$ and $b_{G}$ are $N_{H}\times 1$ and $N_{G}\times 1$ vectors comprising the sensor responses to the homogeneous and gradient fields, respectively.

In theory, the sensor parameters should be obtainable by three homogeneous fields that are not collinear (preferably they are orthogonal) and three gradient fields that span three VSH gradient coefficients. Intuitive explanation for the case where each of the coils generates a distinct VSH component is as follows: Three perfectly homogeneous fields along *x*, *y*, and *z* encode the sensor gain and orientation, as these fields do not depend on the position in space. When the sensor orientation and gain are known, the first-order linear gradients encode the sensor position. As a point of practical interest, six coils in a three-axis Helmholtz configuration would not typically give a robust localization performance. Such a setup can produce the three homogeneous fields, but it can only produce two linear gradients, as just two of $dB_{x}/dx,\;dB_{y}/dy$, and $dB_{z}/dz$ are independent. Thus, a coil producing a transverse gradient (e.g., $dB_{y}/dx$) would be needed.

Even though realistic coils do not produce perfect homogeneous or first-order gradient fields, Equations (5) and (6) provide a good first-order approximation for the sensor parameters in two settings often used in MEG. We now discuss these cases.

*Compensation coil set*: Coil sets that counter external magnetic interference are commonly used in bioelectromagnetic measurements with OPMs (e.g., [19,20]). Typically, the coils in such systems are designed to produce homogeneous and gradient fields; the VSH spectrum of the coil field peaks at a specific low-order component. A good initial estimate for the sensor parameters ($g_{0},\;r_{0})\;$can be obtained by using Equations (5) and (6) with the homogeneous and first-order gradient field coils and their VSH coefficients up to l = 2. The full optimization problem (Equation (2)) can be then solved using the “full” field models (i.e., all VSH coefficients up to the bandlimit $l_{\max}$ given by the limitations of the *a priori* coil field measurement) and $g_{0}$ as well as $r_{0}$ as initial estimates for the sensor parameters. As the low-order VSH coefficients should capture most of the energy of such coil fields, $g_{0}$ and $r_{0}$ should be close to the true sensor parameters. The full optimization then “fine-tunes” the sensor parameters.

*Semi-arbitrary coil arrangement*: The first-order approximation is also useful in the case of a semi-arbitrary coil arrangement (e.g., an arrangement of large square coils). When the VSH coefficients of the *N* coils are known, $B=\left[\;\beta_{1},\ldots ,\;\beta_{N}\right]$, the coil currents i that produce a field with approximately the desired distribution of the VSH coefficients $\tilde{\beta }$ can be obtained with the pseudo-inverse as follows [24]:

(7) $$i=B^{†}\;\tilde{\beta }.$$

When the coil currents are set so that approximately homogeneous fields and first-order gradients are generated, $g_{0}$ and $r_{0}$ can be computed using Equations (5) and (6). Using $g_{0}$ and $r_{0}$ for the initial estimate, the optimization problem (Equation (2)) can be solved using the full VSH models up to $l_{\max}\;$ and the sensor responses to all individual *N* coils.

We note that not all coil arrangements will allow the generation of the desired low-order VSH components. Thus, it might be beneficial to calculate the condition number of matrix B and add coils to the arrangement if the rank is low, and to check the field patterns and the VSH coefficients produced by the currents. As a general guideline for the method to work, we suggest that many coils should be used to cover a large portion of the full solid angle surrounding the sensors, so as to ensure that the coil fields are sufficiently different from one another.

### 2.3. Summary of the Method

In summary, the proposed method for estimating the sensor parameters consists of the following steps:

1. Measure the magnetic fields of the *N* coils with a well-calibrated vector sensor (e.g., fluxgate magnetometer) over the region of the sensor array (the OPMs). Fit VSH models to the measurements.
2. Using the VSH models, compute the currents for exciting homogeneous fields and first-order gradients.
3. Measure the response of the sensor (which is to be calibrated) to the homogeneous fields and gradients.
  - Compute $g_{0}$ and $r_{0}$ using the lowest-order VSH coefficients with Equations (5) and (6).
  - (Optional): Fine-tune the estimates $g_{0}$ and $r_{0}$ by optimizing Equation (2) with full VSH models of the homogeneous and gradient fields.
4. Excite coils individually.
  c. Find the sensor parameters by optimizing Equation (2) using the full VSH models. Use $g_{0}$ and $r_{0}$ (from step 3a or 3b) as initial estimates for the sensor parameters.

## 3. Methods

**Figure 1:**
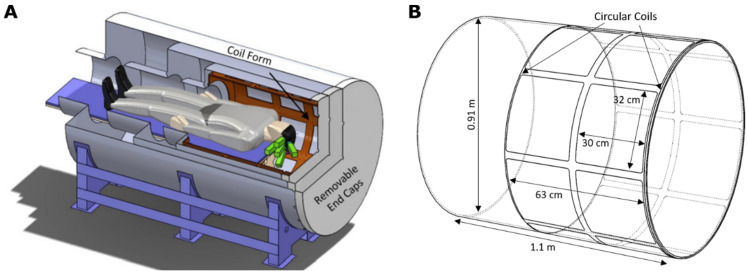
An overview of the OPM–MEG system: (**A**) 3D rendering illustrating the positions of the OPMs (green) inside the shield. Form for the electromagnetic coils is highlighted [18]. (**B**) Schematic illustration of the 16 rectangular and 2 circular coils embedded in the coil form within the shield.

In this section, we outline the methods for calibrating an array of OPM sensors residing inside a magnetic shield. The OPM system is described in detail in Refs. [18,25]; Figure 1 gives an overview of the system. Briefly, the system consists of 6 OPM sensors [26,27], each with 4 channels, within a sensor array holder that can accommodate 9 sensors. Each OPM can measure a magnetic field component on the plane perpendicular to the optical axis of the sensor. With respect to the head, the field components tangential to the scalp are measured. Typically, the two orthogonal tangential components are measured separately. In total, the system has 48 channels (6 sensors with 4 channels measuring 2 field components). The OPMs reside inside a person-sized magnetic shield with 18 embedded electromagnetic coils.

We first measured the magnetic fields of the coils within the sensor array holder using a triaxial fluxgate magnetometer. We then fitted VSH models to these measurements. We validated the calibration method by localizing the fluxgate at different positions within the sensor array holder, as explained in Section 3.3 below. Lastly, we calibrated the OPM array using this method, and utilized the OPM array to localize magnetic dipoles within a phantom.

### 3.1. Measurement of the Coil Fields

We measured the magnetic fields of the 18 coils in the vicinity of the sensor array holder using a three-axis fluxgate magnetometer (FGM3D, SENSYS GmbH, Bad Saarow, Germany). The fluxgate has three channels measuring the orthogonal field components at different locations along the fluxgate axis, with a spacing of 2 cm. The channels are orthogonal within ±0.5°, while their gain is 0.1 V/µT ± 0.1%.

The fluxgate is positioned in the sensor array holder using a custom 3D-printed jig whose dimensions match those of the OPM sensor, so as to ensure a tight fit into the sensor slots in the holder. The jig has four transverse slots for the fluxgate, while the fluxgate position along the longitudinal axis of the jig can be adjusted. For a given fluxgate position, sinusoidal currents of 13.3 mA are sequentially applied to the coils. Altogether we measured 108 locations, giving a total of 324 measurements of the magnetic field of each coil.

VSH coefficients up to the order l = 5 (35 components) were estimated from the measurements using Equation (4). The VSH functions were generated using the Python software package bfieldtools [28,29]. We normalized the VSHs so that each component corresponded to one unit of magnetic energy (“energy” normalization option in bfieldtools). We then set the average coordinates of the measurement positions as the origin of the VSH expansion. This choice of origin was selected to get the zero crossings of the gradient fields into the sensor array volume. We used the root-mean-square (RMS) error normalized by the RMS value of the data to assess the goodness of the VSH fits.

### 3.2. Calibration Methodology

We used the methodology summarized in Section 2.3 to estimate the parameters of the fluxgate magnetometer channels and the OPM sensors. We measured the responses of the sensors (that were to be calibrated) to the coil fields. The coils were excited sequentially using sinusoidal currents with a frequency of 20 Hz. The sensor response amplitudes were estimated from the data using lock-in detection.

Of the 18 shield coils, 17 were used in the measurements, as one of the coils had a broken connector. The 17 coils were driven either individually or in “superposition”, configured to generate the eight first-order VSH fields; the coil currents to generate the first eight VSH components were computed using Equation (7) with the estimated VSH coefficients.

The initial sensor parameter estimates were obtained with Equations (5) and (6) by using the data generated with the first-order VSH fields. The model parameters (the homogeneous field and gradient amplitudes) were estimated from the fitted VSH models. Subsequently, the sensor parameter estimates were “fine-tuned” by optimizing Equation (2) with the first-order VSH field data and the full VSH models. We performed the optimization using the SciPy Python library [30] with the function *minimize*, with default parameters and the Nelder–Mead algorithm. Lastly, the final sensor parameters were obtained by optimizing Equation (2) with the sensor responses to the 17 individual coil fields and full VSH models of the coil fields.

### 3.3. Fluxgate Validation

We positioned the fluxgate sensor within three empty sensor slots of the array (during the time of the measurement six of the nine sensor holders had an OPM inserted). In total, six different positions (two different positions in one sensor slot) were used for the fluxgate, amounting to 18 channels at different positions to calibrate. The currents of the coils were set so that the maximum field amplitude was roughly 12.5 nT over the sensor array holder. We used the proposed method to calibrate the fluxgate channels. The known gains and relative positions/orientations of the fluxgate channels were used to estimate the calibration error of the method.

### 3.4. Calibration of the OPM Array

The OPMs were configured to measure one of the tangential components (denoted as *Bx* or *By*), and the coils were excited individually or in superposition, with a maximum amplitude of 0.5 nT. Altogether, four measurements corresponding to the different sensor/coil configurations (e.g., *Bx* and first-order VSH fields) were performed. The OPM data were recorded using the DAQ system described by Borna et al. in Refs. [18,25]. The data sampling rate of the lock-in amplifier output that served as the OPM magnetometer signal was set to 1 kHz.

The fact that the OPMs we used can measure two components of the magnetic field at the same position motivated us to use two different approaches for the optimization. In the first approach, we optimized the two measurements (*Bx* and *By*) separately, while in the second approach we performed the optimization jointly for the two measurements, by constraining the optimization procedure so that the “two sensors” shared the same position.

We compared the estimated positions of the OPMs to a CAD model of the sensor array by aligning the obtained positions to those of the CAD model using a unit-scale rigid-body transformation obtained with the Umeyama algorithm [31].

### 3.5. Localizing Magnetic Dipoles in a Phantom

We used the OPM array and the obtained sensor parameters to localize small circular coils in a phantom. The 3D-printed phantom had nine hand-wound circular coils with a diameter of 5 mm. The phantom coils were sequentially driven with sinusoidal currents at 20 Hz with an estimated coil dipole moment of about 140 nAm^2^. The amplitudes were extracted from the OPM data using lock-in detection. The measurement was performed independently for *Bx* and *By*; for the dipole localization, the measured amplitudes were combined to give a total of 48 channels.

The coils were localized using the dipole fitting routine in the FieldTrip toolbox (function *ft_dipolefitting;* [32]), with a grid search followed by nonlinear optimization. The coils were modeled as ideal point-like magnetic dipoles. The OPM channels with latency-adjusted time-domain correlation lower than 0.9 with the reference signal fed to the dipole were discarded from the dipole fitting procedure. These channels were deemed unreliable because of the phase offset, likely caused by the OPM cross-axis projection error [33].

Lastly, we compared the estimated positions of the magnetic dipoles to a CAD model of the phantom. To compare their relative positions, the estimated positions were aligned with the CAD model using the Umeyama algorithm [31].

## 4. Results

### 4.1. Modeling the Magnetic Field Measurements with VSHs

**Figure 2:**
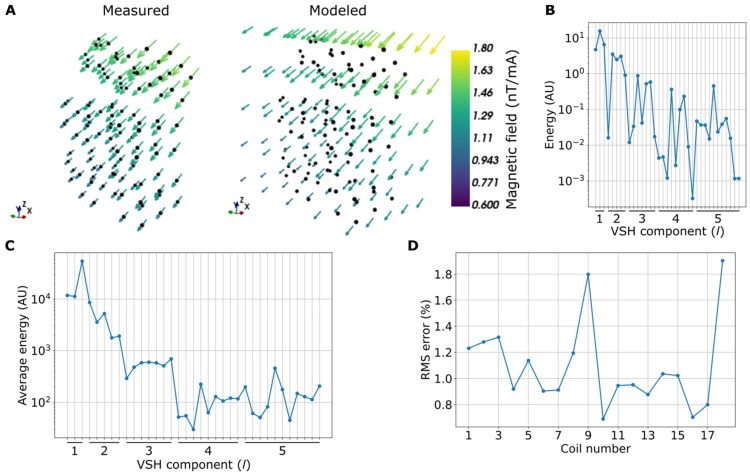
Fitting vector spherical harmonics (VSHs) to the measured magnetic field data: (**A**) Example of a coil magnetic field measured with a three-axis fluxgate at 108 points, together with its VSH fit, extrapolated to a volume surrounding the measurement points. The magnetic field values were normalized by the applied current. (**B**) Magnetic field spectrum of the coil field in panel A given by the squared VSH coefficients. The squared coefficients are shown for each degree *l* and its corresponding *2l + 1* orders. (**C**) Average magnetic field spectrum of the 18 shield coils. (**D**) Normalized RMS errors between the VSH fits and the measurements.

Figure 2 shows the results of fitting the VSH models to the fluxgate measurements. When driving the coils using a current of 13.3 mA, the maximum resulting field amplitudes in the fluxgate measurement points range from 13 to 89 nT, with an average of 24 nT over the 18 coils. Figure 2A shows the measured magnetic field of one of the shield coils, as well as its interpolation using the VSH model. The magnetic field points approximately to the same direction in the measurement volume, with a gradient along the *z*-axis. The normalized RMS error of the VSH reconstruction is 1.0%. The VSH spectrum shown in Figure 2B reveals that the dominant field components are at low orders; the homogeneous components corresponding to l = 1 have the highest energy, followed by the first-order gradients (l = 2). The VSH spectrum averaged over the coils (Figure 2C) confirms that the coils, on average, produce mostly homogeneous and first-order gradient fields. The normalized RMS error is less than 2% for all coils, indicating good fits of the VSH models to the measurements.

**Figure 3:**
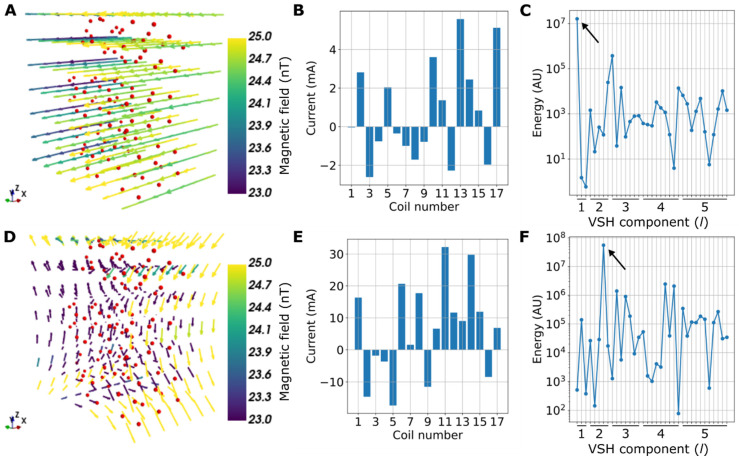
Examples of calculated coil currents of the 17 shield coils that generate specific vector spherical harmonic (VSH) field components: (**A**) Example of a homogeneous magnetic field in the vicinity of the measurement points, shown as red dots. (**B**) The coil currents that generate the field shown in (**A**). (**C**) The VSH spectra of the generated field; the black arrow indicates the energy of the desired VSH component. (**D**–**F**) Same as (**A**–**C**), but for a first-order gradient field.

Figure 3 shows examples of calculated coil currents that approximately generate specific low-order VSH components at the measurement volume. The VSH spectra of the fields peak at specific components, confirming that the currents mainly produce the desired components.

### 4.2. Fluxgate Validation

**Figure 4:**
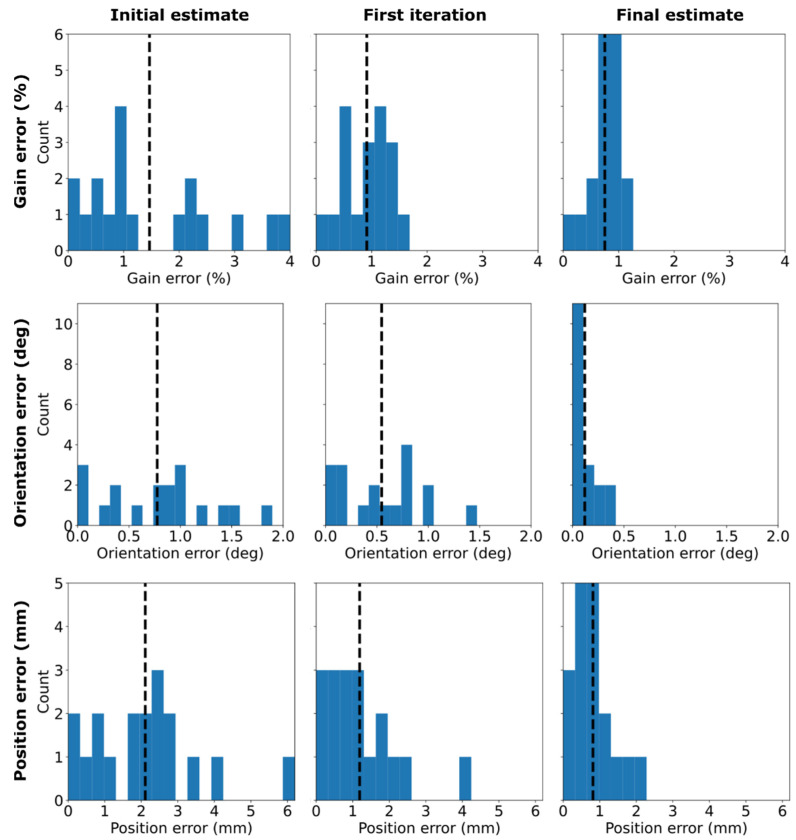
Estimated gain (**top row**), orientation (**middle**), and position (**bottom**) errors for the three channels of the fluxgate magnetometer at six different placements of the fluxgate. Each column of the figure shows the error histograms after different iterations. The initial estimate is given by the matrix pseudo-inversions using the first-order VSH measurements and models. The first-iteration estimate is obtained after fine-tuning the initial estimate to account for the full VSH spectra of the approximate first-order VSH fields. The final estimate is obtained after optimizing the first-iteration estimate with all individual coil fields and their full VSH spectra. The vertical dashed lines indicate the average errors.

Figure 4 shows the estimated fluxgate position, orientation, and gain errors across the six measurement positions (18 channels). Clearly, the errors become smaller as more iterations are performed and as more VSH coefficients are taken into account in the optimization. For the final estimates, the average gain, orientation, and position errors are 0.8% (0.1–1.1%), 0.1° (0.01°–0.4°), and 0.8 mm (0.1–2.0 mm), respectively, while the RMS errors are 0.8%, 0.2°, and 1.0 mm. We estimate that the maximum SNR of this measurement due to the 12.5-nT field at 20 Hz is about 1900, as the average fluxgate noise floor around 20 Hz is 4.6 pT/rt-Hz.

### 4.3. Calibration of the OPM Array and Phantom Localization

**Figure 5:**
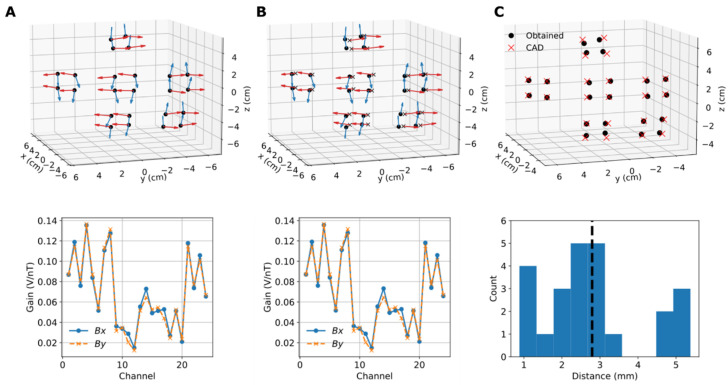
Obtained OPM sensor parameters: (**A**) Sensor parameters (top: position, orientation; bottom: gain) obtained by joint optimization, i.e., assuming that the channels sensing either of the tangential field components (*Bx* or *By*) share the same location. The blue and red arrows correspond to *Bx* and *By*, respectively. (**B**) Sensor parameters obtained by treating the channels separately, i.e., the *Bx* and *By* channels have independent locations. The channel positions for *Bx* and *By* are shown as black dots and crosses, respectively. (**C**) Comparison of the sensor positions in panel A to the CAD model of the sensor array. Histogram illustrates the distances between the positions; vertical dashed line shows the average distance.

The optimized OPM sensor parameters when the OPM channels sensing different field components are treated either jointly or separately are shown in Figure 5A,B, respectively. The sensor positions visually resemble those we expected based on the CAD model of the sensor array, while the obtained orientations are not orthogonal, as also suggested by the characteristics of the magnetic fields of the on-sensor coils (see, e.g., [18]). Interestingly, when the channels sensing *Bx* and *By* are treated separately, they do not localize to the exact same positions. The maximum differences between the parameters obtained jointly or separately are 0.7%, 0.3°, and 4.2 mm for gain, orientation, and position, respectively. The OPMs have an average noise floor of 26 (*Bx*) and 32 fT/rt-Hz (*By*) around 20 Hz; the maximum SNR due to the oscillating 0.5-nT field at 20 Hz is thus approximately 10,000.

Figure 5C compares the jointly obtained sensor positions to those of the CAD model. Altogether, the positions look rather similar; however, the calibrated positions are somewhat more scattered on the plane defined by the sensor-wise channel positions. The calibrated positions for the four channels corresponding to one sensor sit on a single plane quite well. The average distance between the calibrated and CAD positions is 2.8 mm, while the range is 0.9–5.4 mm.

**Figure 6:**
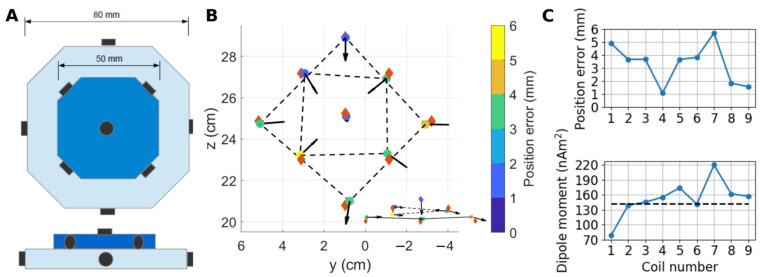
Localization of magnetic dipoles in a phantom using the calibrated 48-channel OPM array: (**A**) A drawing of the 3D-printed phantom showing the locations and orientations of the small circular coils approximating magnetic dipoles (shown black). Both top and side views of the phantom are shown. (**B**) The estimated dipole positions (circles) and orientations of the coil dipole moments (black arrows). Red diamonds show the positions of the coils according to the CAD model of the phantom. The circles are color-coded according to the distance between the estimated position and that of the CAD model. Inset gives a side view of the dipoles. (**C**) The dipole position errors between the CAD model and the estimates, as well as the estimated dipole moments of the coils. The dashed horizontal line shows the dipole moment estimated from the current by which the coils were driven.

The jointly obtained OPM parameters were used to localize magnetic dipoles in a phantom. Figure 6A shows a drawing of the phantom, illustrating the locations of the nine magnetic dipoles. The maximum amplitudes due to the nine dipoles over the OPM channels range from 370 to 1150 pT, with an average of 540 pT. Given the estimated noise levels of the OPM channels, the maximum SNRs across the dipoles range from 10,000 to 30,000. Figure 6B shows the estimated positions and moments of the magnetic dipoles. Visually, the estimated positions correspond well to those of the CAD model, and the estimated orientations of the coil magnetic moments are consistent with the geometry of the phantom. Figure 6C shows the estimated position errors and dipole moments across the dipoles. The average error between the estimated dipole positions and those of the CAD model is 3.3 mm, while the range is 1.1–5.7 mm. On average, the estimated dipole moments differ from those estimated from the current by which the coils were driven by 18%, while the difference can be as large as 54%. The two dipoles with the largest position errors have also the largest errors in the dipole moments.

## 5. Discussion

We proposed a method that uses magnetic fields to find the gain/calibration, position, and orientation of a magnetic field sensor. In particular, we proposed a means to estimate the sensor parameters by using homogeneous and first-order gradient fields (VSHs up to l = 2), which can be generated with specific electromagnetic coils or by driving an appropriate arrangement of multiple individual magnetic field generators. Importantly, if only low-order VSHs are modeled, the sensor parameters can be obtained by simple matrix pseudo-inversions. The sensor parameter estimates can then be enhanced by a more involved optimization, taking into account the higher-order spatial structure of the generated magnetic fields. We especially presented this method in the context of calibrating OPM sensors for MEG.

We showed that the method was able to resolve relative positions and orientations of fluxgate magnetometer channels with RMS errors of 1.0 mm and 0.2°, respectively, while the gain was estimated with an RMS error of 0.8%. Our method also yielded OPM parameters that were close to our expectations; the OPM parameters allowed us to localize magnetic dipoles with an average error of 3.3 mm.

### 5.1. Comparison to Previous Studies

Overall, the requirements for sensor parameter accuracies in (on-scalp) MEG are still not fully understood. Generally, it can be stated that the required accuracy depends on the signal-to-noise ratio and the desired spatial bandwidth (or spatial cutoff frequency) (e.g., [12,34]), as higher spatial frequencies (or “spatial field modes”) are more sensitive to errors. A recent simulation study suggested that in order to obtain similar or higher accuracy with on-scalp MEG than with SQUID–MEG, RMS sensor position and orientation errors should be <4 mm and <10°, respectively [35]. The RMS errors obtained here with the fluxgate magnetometer suggest that the proposed method can fulfill these requirements.

From the fluxgate measurement, we estimate that the position, orientation, and gain errors of the method are roughly below 2.0 mm, 0.4°, and 1.1%, respectively. These are in line with the errors obtained with other recent calibration/localization methods for on-scalp MEG sensors. For example, Pfeiffer et al. (2020) demonstrated errors of less than 2 mm, 3°, and 3%, respectively, with their magnetic method [17], while Gu et al. (2021) estimated that the position and orientation errors of their optical method were less than 1 mm and 0.6°, respectively [14].

Using the calibrated 48-channel OPM array, we were able to localize 5-mm diameter hand-wound circular coils (magnetic dipoles) in a custom phantom, with an average error of 3.3 mm. Using a commercial current dipole phantom (MEGIN Oy, Helsinki, Finland) and optical co-registration of nine OPM sensors/channels (QuSpin Inc., Louisville, CO, USA), Zetter et al. (2019) localized current dipoles with an average error of 2.1 mm [11]. More recently, Boto et al. (2022) combined non-synchronous triaxial OPM measurements (25 sensors; 75 channels; QuSpin Inc.) to localize current dipoles in a custom phantom with an error of 5.2 mm [36]. Our dipole localization error is thus similar to what has been achieved in previous OPM studies.

### 5.2. Study Design and Caveats

For both fluxgate (Section 4.2.) and OPM (Section 4.3.) measurements, we chose the field amplitudes to maximize the SNR. For the fluxgate, 12.5 nT was roughly the maximum amplitude such that the currents that excite the homogeneous and gradient fields are implemented without saturating the coil drivers. For OPMs, 0.5 nT was used to maximize the SNR within the limits of the OPM linear range (approximately ±1 nT). Moreover, with the OPMs, we wanted to use as high a field amplitude as possible, so as to reduce the effects caused by the finite bit resolutions of the coil drivers’ digital-to-analog converters. The digital-to-analog converters may cause quantization noise when implementing currents for the homogeneous and gradient field components (Figure 3).

Our method is based on accurate measurement of the coil fields using a well-calibrated magnetic sensor. For this task, we used a fluxgate magnetometer, whose datasheet specifies the relative positions, orientations, and gains of the channels, as well as their errors. Systematic errors and thermal drifts in the fluxgate parameters ultimately limit the accuracy at which we can calibrate the OPMs using our method. Moreover, the spatial low-pass filtering by the fluxgate may limit the accuracy at which higher-order VSH coefficients can be estimated if the sensitive volume of the fluxgate channel becomes comparable to the spatial frequency of the VSH component. We further note that scaling of the VSH models may cause additional errors if the models do not scale linearly from the fluxgate field amplitude range (~10 nT) to the OPM range (below 1 nT).

We lack the ground truth against which to compare the OPM sensor parameters. For example, we expect that the CAD OPM positions do not perfectly reflect the real channel positions. The CAD model uses the center of the laser beam profile in the vapor cell as the channel position. However, the “magnetic center-of-mass” of the channel is defined by the spin polarization profile in the cell. It is our understanding that the spin polarization is not homogeneous in the vapor cell, and is larger on the side of the cell where the pump laser beam enters the cell, shifting the center of mass of the channel towards the cell wall. Thus, the distances between the CAD model and the obtained positions shown in Figure 5C probably overestimate the position errors.

Ultimately, the OPM response nonlinearities—such as the cross-axis projection error [33]—may limit the accuracy at which the OPM sensor parameters can be obtained with methods based on magnetic fields. However, as shown here, these errors may be alleviated to some degree by constraining the optimization, by combining multi-axis measurements. The sensor parameters obtained by using combined channel positions had smaller average dipole localization error than those obtained with separate channel positions (3.3 vs. 4.0 mm; results not shown here). Additionally, the bit resolution of the digital-to-analog converters driving the coils may have limited how accurately we could implement the currents to generate the low-order VSH components (Figure 3) for the OPM sensors, reducing the localization accuracy compared to the fluxgate magnetometer.

### 5.3. Future Directions

The proposed method gives OPM positions and orientations in a common coordinate frame. Additional steps are needed to co-register the OPMs with the subject’s head and, ultimately, with MRI images. To accomplish that, digitized head-position indicator coils could be localized with the OPM array, as is done traditionally with SQUID–MEG systems. In addition, the coordinate system given by this method contains the field models of the coils, which can be used to remove interference caused by the coils from the OPM data.

With the low-order field models, the sensor parameters can be obtained by pseudo-inversion of two low-dimensional matrices. Due to the small number and low computational cost of the operations needed, this method might be well suited for fast real-time tracking of the sensor parameters. Such tracking may be accomplished by feeding sinusoids at different frequencies to the coils, with subsequent lock-in detection of the coil amplitudes. For this approach, a frequency band must be reserved for the coil signals; the required bandwidth will depend on the number of coils used and the desired time resolution. For the tracking approaches, it may be beneficial to first run the full optimization routine (Section 2.3) using static sensors to determine the sensor parameters as accurately as possible. For real-time tracking, the sensor parameters could be updated with the first-order model. The model could also incorporate, for example, some priors based on how fast the sensor parameters are likely to change.

We used a comprehensive fluxgate measurement of the coil magnetic fields to determine their VSH field models. We note that the fluxgate measurement of the coil fields was performed in 2019, while the validation with the fluxgate and calibration of the OPM array were carried out in 2021. It seems that the coil VSH models stayed quite accurate, even though the shield was opened and degaussed numerous times between 2019 and 2021. There are three compelling alternatives to the comprehensive but rather cumbersome fluxgate measurement: First, the fluxgate measurement positions could be optimized to yield the desired VSH coefficients with as low a number of measurements as possible. Second, with optical tracking of the fluxgate position, a large number of measurements of the coil fields could be collected rather quickly (see Refs. [37,38]). The spatial oversampling of the coil fields could reduce the effects of random fluxgate positioning errors on the obtained VSH coefficients. Lastly, instead of treating the sensors (to be calibrated with the method) independently, the whole array could be treated jointly to leverage the spatial sampling provided by the array to directly estimate the VSH coefficients and sensor parameters from the excited coil fields, without a separate *a priori* measurement of the coil fields. This approach would probably benefit from a large number of sensors in the array.

## 6. Conclusions

We proposed a method that uses external magnetic field generators to localize a magnetic field sensor by minimizing the overall error between the field models of the generators and the measurements by the sensor. We modelled the magnetic fields of the generators using vector spherical harmonics based on *a priori* measurements of the fields. To simplify the computations in method, we proposed a way to estimate the gain, position, and orientation of a magnetic sensor by using homogeneous and first-order gradient fields; we further showed how these estimates can be enhanced with nonlinear optimization. This method allowed us to estimate the relative positions, orientations, and gains of the channels of a fluxgate magnetometer with RMS errors of 1.0 mm, 0.2°, and 0.8%, respectively. We used this method to calibrate a 48-channel optically pumped magnetometer array; the calibrated array allowed us to localize magnetic dipoles with an average error of 3.3 mm. Our results demonstrate the feasibility of using this method to calibrate and localize optically pumped magnetometers for magnetoencephalography.

## Data Availability

The scripts for sensor array calibration are available from the corresponding author upon reasonable request.

## Appendix A

Here, we derive Equations (5) and (6) for obtaining the sensor parameters with the first-order VSH models. The vector sensor gain is $\vec{g}=g\hat{n}=g_{x}{\hat{e}}_{x}+g_{y}{\hat{e}}_{y}+g_{z}{\hat{e}}_{z}$, where hat denotes unit vector and ${\hat{e}}_{x}$ denotes the unit vector along the *x*-axis. Supposing that we have a homogeneous field ${\vec{B}}_{i}=H_{i,x}{\hat{e}}_{x}+H_{i,y}{\hat{e}}_{y}+H_{i,z}{\hat{e}}_{z}$, the sensor response to the field is as follows:

(A1) $$b_{i}=\vec{g}\cdot {\vec{B}}_{i}=H_{i,x}g_{x}+H_{i,y}g_{y}+H_{i,z}g_{z}.$$

If we have a collection of these equations, they can be written in the matrix form as follows:

(A2) $$b_{H}=Hg,$$

where the elements of the vectors and matrices are defined as $b_{H}\left[i\right]=b_{i}$, $H\left[i,j\right]=H_{i,j}$, $g\left[j\right]=g_{j}$, and j∈{x,y,z}, where the index-to-coordinate mapping is as {x:1, y:2, z:3}. The sensor gain can then be solved from Equation (A2) using the pseudo-inverse, as shown in Equation (5). Please note that we use the pseudo-inverse because the matrix H is not necessarily square.

Next, we model the field of the *i*th gradient coil in a matrix form as follows:

(A3) $$\left(\begin{array}{c} B_{x,i}\\ B_{y,i}\\ B_{z,i} \end{array}\right)=\left(\begin{array}{ccc} G_{xx,i} & G_{yx,i} & G_{zx,i}\\ G_{yx,i} & G_{yy,i} & G_{yz,i}\\ G_{zx,i} & G_{yz,i} & -G_{xx,i}-G_{yy,i} \end{array}\right)\left(\begin{array}{c} x\\ y\\ z \end{array}\right)+\left(\begin{array}{c} B_{x,i}^{H}\\ B_{y,i}^{H}\\ B_{z,i}^{H} \end{array}\right)\Leftrightarrow B_{i}=G_{i}r+B_{i}^{H},\;$$

where the elements of the gradient matrix $G_{i}$ are defined as Gxy,i=dBx,idy, and $B_{x,i}^{H}$ are the homogeneous components of the gradient field. The gradient matrix is defined so that its elements satisfy Maxwell’s equations in a source-free space $\nabla \cdot \vec{B}\;=\;0$ and $\nabla \;\times \;\vec{B}\;=\;0$, i.e., the sum of its diagonal elements must be zero ($G_{xx,i}$ + $G_{yy,i}\;-\;G_{xx,i}\;-\;G_{yy,i}\;=\;0$), and it has to be symmetrical ($G_{xy,i}\;=\;G_{yx,i}).$ In other words, two of the three diagonal gradients are independent, while three off-diagonal gradients are independent. A measurement of the *i*th gradient coil can be written as follows:

(A4) $$b_{i}\;=\;\vec{g}\cdot {\vec{B}}_{i}\;=\;\left(G_{i}\left[1,:\right]g\right)x\;+\;\left(G_{i}\left[2,:\right]g\right)y\;+\;\left(G_{i}\left[3,:\right]g\right)z\;+\;{\left(B_{i}^{H}\right)}^{T}g,$$

where $G_{i}\left[j,:\right]$ denotes the *j*th row of the gradient matrix of the *i*th coil. A collection of the gradient coil measurements can then be written as follows:

(A5) $$b_{G\;}=Gr+H_{G}g,$$

where the matrix elements are defined as $b_{G}\left[i\right]=b_{i},\;G\left[i,j\right]=G_{i}\left[j,:\right]g$**,** and $H_{G}\left[i,:\right]={\left(B_{i}^{H}\right)}^{T}$. Equation (A5) can then be solved to yield Equation (6).
